# Dual roles of extracellular vesicles in acute lymphoblastic leukemia: implications for disease progression and theranostic strategies

**DOI:** 10.1007/s12032-024-02547-7

**Published:** 2024-11-22

**Authors:** Mahya Sadat Lajevardi, Mahshad Ashrafpour, Shaden M. H. Mubarak, Behnoosh Rafieyan, Arash Kiani, Effat Noori, Marzieh Roayaei Ardakani, Maryam Montazeri, Niloofar Kouhi Esfahani, Naghmeh Asadimanesh, Saeed Khalili, Zahra Payandeh

**Affiliations:** 1 Department of Medical Biotechnology, School of Medicine, Zanjan University of Medical Sciences, Zanjan, Iran; 2 School of Health Sciences, University of Georgia, Tbilisi, Georgia; 3 Department of Clinical Laboratory Science, Faculty of Pharmacy, University of Kufa, Kufa, Iraq; 4 School of Medicine, Semnan University of Medical Sciences, Semnan, Iran; 5 Student Research Committee, Yasuj University of Medical Sciences, Yasuj, Iran; 6 Department of Biotechnology, Faculty of Medicine, Shahed University, Tehran, Iran; 7 Faculty of Medicine, Jondishapour University of Medical Sciences, Ahvaz, Iran; 8 Razi Clinical Researches Development, Mazandaran University of Medical Science, Sari, Iran; 9 Faculty of Medicine, People’s Friendship University of Russia (Rudn University), Moscow, Russia; 10 School of Medicine, Shahid Beheshti University of Medical Sciences, Tehran, Iran; 11 Department of Biology Sciences, Shahid Rajaee Teacher Training University, Tehran, 1678815811 Iran; 12 Department of Rheumatology and Inflammation Research, Institute of Medicine, Sahlgrenska Academy, University of Gothenburg, 41346 Gothenburg, Sweden

**Keywords:** Acute lymphocytic leukemia, Extracellular vesicles, MiRNAs, Immunotherapy

## Abstract

Acute Lymphoblastic Leukemia (ALL) is a heterogeneous blood cancer characterized by the uncontrolled growth of immature lymphoid cells due to dysregulated signaling pathways. It is the most common pediatric cancer, with high cure rates in children, but significantly lower survival rates in adults. Current theranostic strategies, including chemotherapy, immunotherapy, and nanomedicine, aim to improve detection and treatment precision but are limited by side effects, drug resistance, high costs, and stability issues. Notably, extracellular vesicles (EVs) offer a promising alternative, addressing these limitations through their natural biocompatibility and targeted delivery capabilities. EVs play a dual role in ALL: they contribute to leukemia progression by promoting tumor growth, immune suppression, and drug resistance via the transfer of oncogenic molecules, while also serving as valuable non-invasive biomarkers due to their specific miRNA and protein content. Their ability to deliver therapeutic agents directly to leukemic cells, combined with their stability and low immunogenicity, makes EVs a compelling tool for improving ALL treatments. Indeed, by targeting the molecular pathways influenced by EVs or leveraging them for drug delivery, innovative therapeutic strategies can be developed to enhance treatment outcomes and reduce side effects. Thus, EVs represent a promising frontier for advancing theranostic strategies in ALL, offering new opportunities to improve diagnosis and treatment while overcoming the limitations of traditional therapies. This review will explore the dual roles of EVs in ALL, addressing their contributions to disease progression and their potential as therapeutic agents and biomarkers for early diagnosis and targeted therapies.

## Introduction

Leukemia is a malignant condition originating from abnormal bone marrow hematopoietic stem cells, encompassing a range of disorders, such as chronic myeloid leukemia (CML), chronic lymphocytic leukemia (CLL), acute myeloid leukemia (AML), and acute lymphocytic leukemia (ALL) [1]. Acute leukemia is a leading cause of cancer-related deaths among young adults, with low survival rates. Theranostic strategies for ALL combine chemotherapy, immunology, nanomedicine, and aptamers to enhance detection and treatment. However, these approaches are limited by side effects, drug resistance, costs, immune reactions, and stability issues. Despite progress in treatment, relapse rates remain high, resulting in a 5-year survival rate of only 40% to 50% [2–5]. Therefore, it is crucial to unravel the complexities of leukemia causation, metastasis dynamics, and factors triggering relapse, with a significant focus on discovering novel strategies for detecting and treating ALL.

A novel strategy involves using extracellular vesicles (EVs) in ALL treatment, as they exhibit a dual role: Advancing the disease while also offering significant therapeutic and diagnostic benefits. On the pathologic side, EVs promote tumor growth by carrying oncogenic molecules like microRNAs (miRNAs), proteins, and DNA, which alter the bone marrow microenvironment to support leukemic cell proliferation. For instance, miRNAs, which can function as tumor suppressors (e.g., miR-15, miR-16, let-7) or oncogenes (e.g., miR-155, miR-17-92, miR-21), significantly influence leukemia; dysregulation of these molecules contributes to leukemia’s initiation and progression, making them promising therapeutic targets [6–10]. They also facilitate drug resistance by transferring molecules that upregulate drug-resistant proteins, protecting leukemia cells from chemotherapy. Additionally, EVs help leukemic cells evade the immune system by modulating immune cell functions and creating an immunosuppressive environment [7–10].

On the therapeutic side, EVs can serve as biomarkers for early detection and prognosis of ALL. Indeed, they serve as biomarkers for early detection and prognosis due to their specific molecular cargo, like proteins and miRNAs. EVs can be engineered to deliver therapeutic agents directly to leukemia cells, enhancing treatment efficacy and specificity. Additionally, EV-based immunotherapies are being developed to activate immune cells to target leukemia or directly modify immune cell behavior. These approaches provide targeted therapy and strengthen the immune response against leukemia, making EVs versatile tools in fighting ALL [10–12].

This review will examine the dual roles of EVs in ALL, focusing on their contributions to disease progression and their potential as therapeutic agents. It will discuss mechanisms of tumor growth, drug resistance, and immune evasion, while highlighting their clinical applications as biomarkers for early diagnosis and targeted therapies.

## Current theranostic strategies in ALL

The treatment landscape for adult ALL has significantly evolved, incorporating a variety of theranostic strategies. However, inherent limitations in each approach highlight the urgent need for innovative alternatives, such as the use of EVs.

Conventional chemotherapy has long been the cornerstone of ALL treatment, capable of inducing remission in many patients. Despite this, the overall cure rates remain disappointingly low, especially in adults, due to the increased toxicity and complications associated with aging. Long-term survival rates are often below 40% [13]. Targeted therapies have emerged as a promising complement to chemotherapy. For example, monoclonal antibodies like rituximab and inotuzumab ozogamicin have improved outcomes for patients with specific B-cell immunophenotypes. Nevertheless, these therapies primarily target B-cell ALL and may not be effective for T-cell ALL or other subtypes [14–16]. Additionally, tyrosine kinase inhibitors (TKIs) have transformed the treatment for Philadelphia chromosome-positive ALL, significantly enhancing survival rates. However, resistance to these agents can develop, limiting their universal applicability across all ALL patients [17].

Immunotherapy has introduced innovative strategies, including chimeric antigen receptor (CAR) T-cell therapy, which has shown great promise, particularly in relapsed or refractory cases. Yet, CAR-T-cell therapy is associated with severe side effects, such as cytokine release syndrome and neurotoxicity, which can limit its use in older adults or those with comorbidities [18–20]. Similarly, bispecific antibodies have demonstrated effectiveness in targeting cancer cells, particularly leukemia cells, but carry risks of severe adverse events, complicating their implementation in clinical settings [20, 21].

In recent years, nanomedicine and EVs have emerged as promising avenues for enhancing treatment outcomes in ALL. Nanomedicine focuses on advanced drug delivery systems and diagnostics, potentially improving therapeutic efficacy through targeted delivery mechanisms [22]. EVs, as natural nanoparticles involved in intercellular communication, can also be engineered for the precise delivery of therapeutic agents directly to leukemia cells [23]. Both nanomedicine and EVs offer opportunities to improve treatment specificity and efficacy while minimizing systemic toxicity. However, challenges related to biocompatibility, production complexity, and regulatory hurdles remain significant barriers to their widespread application [22]. Aptamer-based therapies represent another promising alternative, leveraging their specificity for leukemia cells to enhance treatment precision. While still in the early stages of clinical application, further research is essential to establish their efficacy and safety in treating ALL [24].

On the whole, the integration of advanced therapeutic strategies—including immunotherapy, nanomedicine, and EVs—offers exciting potential for improving treatment outcomes in adult ALL. These innovative approaches aim to enhance the specificity and efficacy of therapies, addressing the limitations of traditional treatment methods and providing hope for better management of this challenging disease [2, 3, 25–27]. (Table 1).

**Table 1:** Conventional strategies in ALL

Category	Therapy	Mechanism/application	References
Current treatments	Chemotherapy	Disruption of cell cycle, induction of cell death	[28]
	Radiation therapy	Control of CNS recurrence	[29]
	Allogeneic HSCT	For high-risk patients in first complete remission (CR1)	[30]
Passive immunotherapy	Monoclonal antibodies		
	Rituximab (anti-CD20)	Induces apoptosis and activates complement-mediated cytotoxicity	[31]
	Daratumumab (anti-CD38)	Induces apoptosis via multiple mechanisms	[32]
	Isatuximab (anti-CD38)	Directly targets CD38 to induce apoptosis	[33]
	Epratuzumab (anti-CD22)	Recruits immune cells through Fc receptors	[34]
	IL-7Rα (CD127)	Induces NK-mediated T-ALL cell death	[35]
	Alemtuzumab (anti-CD52)	Induces apoptosis and cell-mediated cytotoxicity	[36]
Antibody–drug conjugates	ADCT-602 (anti-CD22)	Exhibits anti-tumor activity	[37]
	ADCT-402 (anti-CD19)	Dose-dependent anti-tumor activity against CD19 + malignancies	[38]
	SGN-CD19A (anti-CD19)	Induces anti-tumor activity	[39]
	Inotuzumab ozogamicin (anti-CD22)	Induces apoptosis in CD22 + B-cell lymphoma	[40]
Bispecific antibodies (BiTEs)	Blinatumomab (anti-CD19xCD3)	Favorable toxicity profile	[41]
	Vibecotamab (anti-CD3xCD123)	Recruits cytotoxic T cells to kill CD123 + tumor cells	[42]
Aptamers	Sgc8 (anti-PTK7, various payloads)	Enhances cytotoxicity and inhibits cell growth	[43–45]
CAR-T-cell therapy	Various CAR-T therapies	MHC-unrestricted killing of tumor cells through various mechanisms	[46–55]
CAR-NK cells	CD19, CD5, CD7, CD4 CAR-NK cells	Target specific antigens, leading to cytokine secretion and tumor lysis	[56–59]
Nanoplatforms (miscellaneous)	Various nanoparticle formulations	Enhance cytotoxicity and anti-leukemic efficacy	[60–76]

## Roles of EVs in ALL

EVs have emerged as a significant focus in recent leukemia research due to their pivotal roles in disease initiation and progression [27, 77, 78]. These small vesicles, released by cancer cells, transport a wide array of biomolecules, including proteins, miRNAs, and double-stranded DNA (dsDNA). The molecular content of EVs plays a profound role in leukemia pathogenesis, particularly within the bone marrow microenvironment (BMM), acting as key mediators of intercellular communication [79]. This interaction influences various cellular processes critical to the disease, highlighting the importance of EVs in shaping the leukemic landscape.

One of the critical functions of EVs is promoting angiogenesis, the formation of new blood vessels that supply tumors with essential nutrients and oxygen. This process is vital for tumor growth, as larger and more aggressive leukemias require an enhanced blood supply to sustain their metabolic demands. EVs not only deliver metabolic substrates and enzymes that support the survival of leukemic cells but also carry signaling molecules that stimulate cell proliferation. Consequently, these vesicles accelerate tumor growth, contributing to the aggressive nature of leukemia [80, 81].

EVs are also instrumental in the development of drug resistance, a significant challenge in leukemia treatment. They can transfer specific miRNAs that either suppress tumor suppressor genes or upregulate proteins linked to resistance mechanisms [81]. For example, certain miRNAs can inhibit the expression of pro-apoptotic genes, allowing leukemic cells to evade programmed cell death even in the presence of chemotherapeutic agents. This transfer of resistance mechanisms complicates treatment strategies, necessitating the exploration of novel interventions that target EVs to overcome resistance [82].

Moreover, EVs actively remodel the bone marrow niche, fostering an environment that supports the survival and proliferation of leukemic cells while suppressing immune responses. Thus, by altering the composition and activity of the BMM, EVs contribute to an immunosuppressive microenvironment that enables leukemic cells to evade detection and destruction by the immune system. This capacity to modulate immune responses is critical, particularly as it impedes the effectiveness of immunotherapies designed to activate the body’s immune system against cancer [83].

Nonetheless, the ability of EVs to carry genetic and epigenetic abnormalities makes them valuable for clinical applications [84]. For instance, profiling specific miRNAs in EVs may help identify patients at a higher risk of relapse or treatment failure, enabling clinicians to tailor therapeutic approaches accordingly [85]. Furthermore, the detection of unique EV markers in bodily fluids, such as blood or bone marrow, could provide insights into disease burden or treatment response, facilitating improved risk stratification. Indeed, by identifying specific genetic signatures within EVs, clinicians can better understand the molecular underpinnings of leukemia and make more informed decisions regarding patient management [81, 86].

**Fig. 1:**
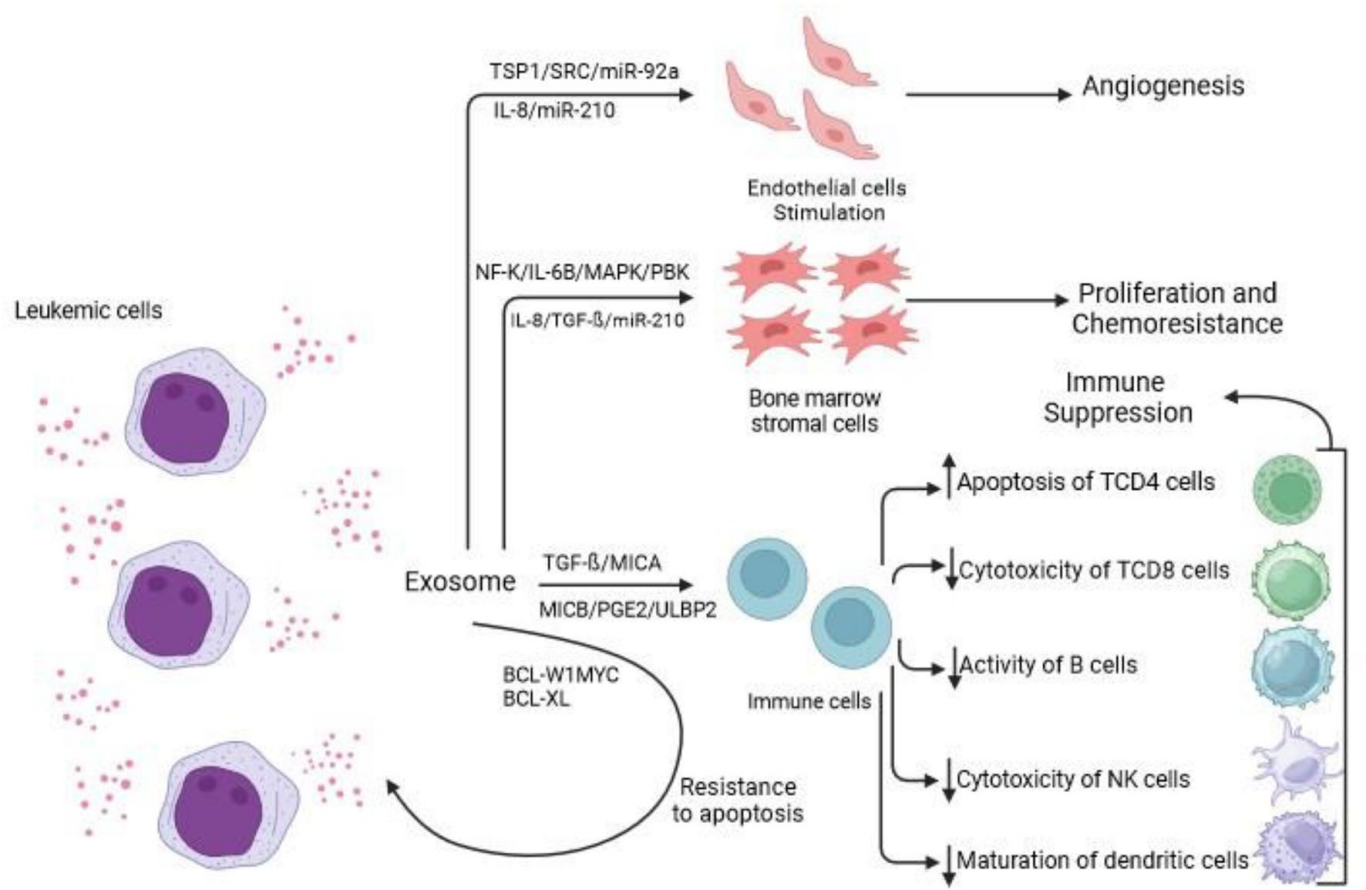
The role of EVs in the pathogenesis and progression of ALL. In the context of ALL, EVs derived from leukemic cells are shown to influence various aspects of disease biology, such as leukemic cell proliferation, drug resistance, immune evasion, and communication with the bone marrow microenvironment. Additionally, EVs derived from stromal cells and other components of the TME are illustrated to contribute to disease progression by modulating leukemic cell behavior and altering the surrounding niche

In ALL, harnessing EVs for biomarker identification presents promising opportunities for enhancing diagnosis and personalizing therapy [78, 87]. For example, the detection of specific EV-derived miRNA profiles could help differentiate between various ALL subtypes, guiding treatment decisions [88]. Besides, utilizing blood samples to assess EV content offers a less invasive alternative to traditional bone marrow biopsies for monitoring disease status over time [86]. Overall, the integration of EV profiling into clinical practice holds great potential for advancing the understanding and management of leukemia, ultimately leading to improved patient outcomes through more effective and personalized therapeutic strategies (Fig. 1).

### Regulatory impact of EVs on the BMM

Leukemia-derived EVs have emerged as pivotal modulators of the BMM in ALL. These small membrane-bound vesicles carry a diverse array of bioactive molecules, including proteins, miRNAs, and other signaling molecules, that significantly impact the behavior of stromal and immune cells within the bone marrow [27, 78, 81, 89].

One of the key mechanisms through which EVs influence the BMM is by transferring oncogenic miRNAs into bone marrow mesenchymal stromal cells (BM MSCs). For instance, two oncogenic miRNAs, miR-155 and miR-181, are frequently upregulated in various types of leukemia. When leukemic cells release EVs containing these miRNAs, they can fuse with or be taken up by BM MSCs, leading to the alteration of the recipient cells’ gene expression profiles. This transfer is a critical mechanism by which leukemic cells manipulate the surrounding microenvironment to support their own survival and proliferation [90].

Once inside the BM MSCs, miR-155, and miR-181 suppress the expression of crucial regulatory genes like SOCS1 and PTEN. SOCS1 (Suppressor of Cytokine Signaling 1) is a negative regulator of cytokine signaling, specifically in pathways, such as JAK/STAT, which are activated by various growth factors and cytokines. Indeed, by inhibiting SOCS1, the BM MSCs become more responsive to cytokines, resulting in enhanced signaling that promotes leukemic cell survival. On the other hand, PTEN (Phosphatase and Tensin Homolog) is a well-known tumor suppressor that counteracts the PI3K/AKT pathway, which is critical for cell survival, growth, and proliferation. Downregulation of PTEN, therefore, diminishes its regulatory influence, leading to increased activation of pro-survival signaling pathways [27, 78, 91].

The downregulation of SOCS1 and PTEN alters the secretion profile of BM MSCs, significantly increasing the production of pro-survival molecules, particularly interleukin-6 (IL-6) and stromal-derived factor 1 (SDF-1 or CXCL12). IL-6 is a pleiotropic cytokine that plays a vital role in inflammation and immune responses. Elevated levels of IL-6 in the bone marrow microenvironment contribute to a chronic inflammatory state that not only supports leukemic cell growth but also disrupts normal hematopoiesis by impairing the functionality of healthy HSCs. Specifically, IL-6 can inhibit the differentiation of HSCs into mature blood cells, allowing leukemic cells to dominate and outcompete normal hematopoiesis [92, 93].

CXCL12, also known as SDF-1, is another critical factor produced by BM MSCs in response to the altered signaling landscape created by EV-derived miRNAs. CXCL12 enhances the retention and homing of leukemic cells within the bone marrow. It does so by binding to its receptor, CXCR4, on leukemic cells, promoting their migration and retention in the bone marrow niche. This interaction fosters a protective microenvironment for leukemic cells, shielding them from therapeutic interventions and further supporting their proliferation and survival [94].

EVs disrupt normal hematopoiesis by directly targeting hematopoietic stem cells (HSCs) and progenitor cells, as seen with EVs carrying miR-486, which interfere with key differentiation pathways and inhibit healthy blood cell production [95]. This disruption allows leukemic cells to outcompete their normal counterparts for resources and space within the bone marrow, contributing to the cytopenias frequently observed in leukemia patients; by altering the balance between leukemic and healthy cells, EVs effectively tilt the microenvironment in favor of leukemia, facilitating its dominance [96]. Additionally, EVs play a critical role in remodeling the extracellular matrix (ECM) of the BMM by carrying matrix metalloproteinases (MMPs), enzymes that degrade ECM components and create physical space for leukemic cells to invade and establish themselves [97]. This degradation alters the structural integrity of the bone marrow and releases additional growth factors, such as transforming growth factor-beta (TGF-β), which further stimulates leukemic growth and survival, ultimately establishing a more favorable microenvironment for their persistence while facilitating leukemic cell expansion [98].

**Fig. 2:**
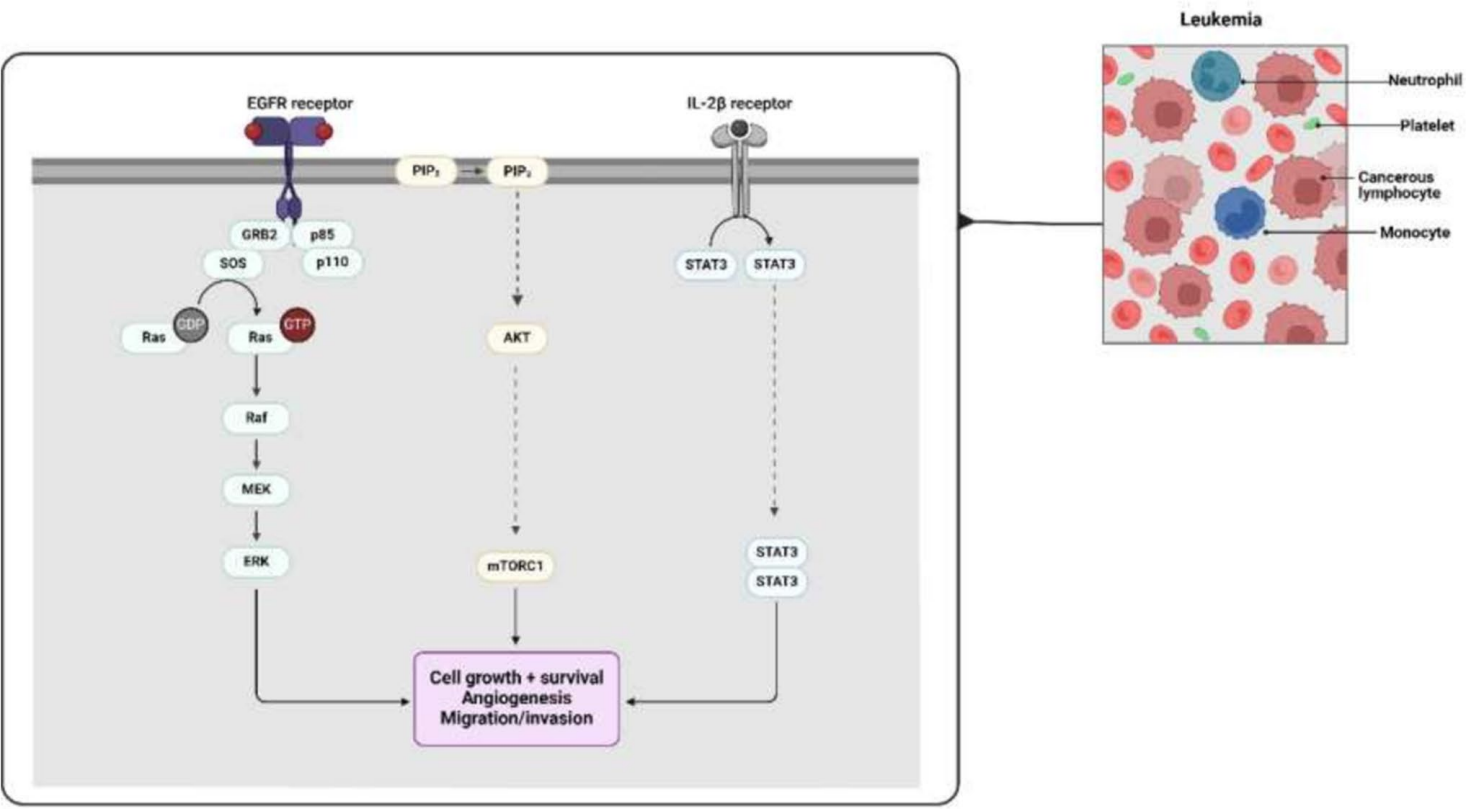
In ALL, several key molecular pathways contribute to cancer progression, invasion, and metastasis. The EGFR (epidermal growth factor receptor) pathway is activated upon ligand binding, triggering downstream signaling cascades, such as the Ras/Raf/MEK/ERK and PI3K/AKT/mTOR pathways. These cascades promote cell survival, proliferation, and angiogenesis. Specifically, AKT and mTOR regulate cell growth and metabolism, while ERK influences cell division. STAT3 is activated by cytokines like IL-2β, which promotes cell survival and immune evasion. AMPK, a metabolic sensor, can negatively regulate mTOR to limit excessive growth, but its dysregulation contributes to cancer progression. Together, these pathways drive leukemia cells’ ability to grow uncontrollably, invade surrounding tissues, and metastasize, making them critical therapeutic targets

EVs derived from leukemia cells are pivotal in promoting angiogenesis, a process crucial for the progression of leukemia. They achieve this by delivering key pro-angiogenic factors to endothelial cells within the bone marrow. Among these factors, VEGF plays a central role. VEGF binds to its receptors on the surface of endothelial cells, triggering a cascade of signaling pathways, notably the PI3K/AKT, MAPK/ERK, and JAK/STAT pathways. The activation of the PI3K/AKT pathway enhances cell survival and growth, which is essential for the stability and longevity of newly formed blood vessels [99]. Simultaneously, the MAPK/ERK pathway is responsible for driving endothelial cell proliferation and migration, processes that are integral to the formation of new blood vessels. The cooperation of these pathways facilitates not only the structural formation of blood vessels but also ensures their functionality, which is necessary to supply nutrients and oxygen to the expanding leukemic mass [99] (Fig. 2).

In addition to VEGF, miR-210 is another critical pro-angiogenic factor transported by leukemic EVs. This miRNA plays a significant role under the hypoxic conditions frequently encountered within tumors. miR-210 stabilizes hypoxia-inducible factor 1-alpha (HIF-1α), a transcription factor that induces the expression of various pro-angiogenic genes, including VEGF itself. By upregulating these genes, miR-210 promotes angiogenesis, ensuring that the tumor microenvironment remains adequately vascularized. Furthermore, miR-210 also targets and downregulates Ephrin-A3 (EFNA3), an anti-angiogenic factor that typically inhibits blood vessel formation. Thus, miR-210 enhances the angiogenic potential of endothelial cells, thereby supporting the growth and survival of leukemic cells through improved nutrient and oxygen delivery [100, 101].

Beyond their role in angiogenesis, leukemia-derived EVs also play a crucial role in modulating immune responses, thereby facilitating leukemia progression. These EVs carry immunosuppressive molecules, such as transforming growth factor-beta (TGF-β) and programmed cell death ligand 1 (PD-L1), which directly influence the activity of immune cells within the bone marrow microenvironment [102]. TGF-β is known for its capacity to impair T-cell proliferation and function, effectively diminishing the immune system’s ability to mount a robust response against leukemic cells. On the other hand, PD-L1 binds to PD-1 receptors on T cells, inhibiting their activation and further reducing their capacity to recognize and eliminate leukemia cells. This interaction between PD-L1 and PD-1 is a critical checkpoint mechanism that tumors exploit to evade immune detection [102].

Moreover, EVs derived from leukemic cells can suppress the activity of natural killer (NK) cells, which are essential components of the innate immune system responsible for targeting and killing malignant cells. Leukemia-derived EVs can limit the secretion of cytotoxic granules, such as perforin and granzymes, from NK cells, thereby diminishing their ability to effectively eliminate leukemic cells [103]. In addition to inhibiting cytotoxicity, these EVs promote the expansion of regulatory T cells (Tregs). Tregs are immunosuppressive cells that dampen immune responses by inhibiting the activity of effector T cells, further fostering a tolerogenic environment that allows leukemia cells to thrive [104].

The collective immunosuppressive effects of leukemic EVs enable leukemia cells to evade immune surveillance effectively. This immune evasion contributes to disease progression and enhances the resistance of leukemia cells to immune-based therapies, making it challenging to achieve successful treatment outcomes [105]. Indeed, by remodeling the bone marrow microenvironment, promoting angiogenesis, and suppressing immune responses, leukemia-derived EVs create a supportive niche that facilitates leukemia progression and complicates therapeutic interventions [106]. Thus, targeting these EV-mediated pathways holds promise for developing novel therapeutic strategies aimed at disrupting the supportive microenvironment of leukemia and enhancing anti-leukemic immune responses.

### Effects of EVs on leukemia cell proliferation

EVs significantly influence the proliferation and apoptosis of leukemia cells through complex mechanisms involving the transfer of specific miRNAs and other signaling molecules [107]. In ALL, for instance, studies have shown that miRNA-181b-5p is highly expressed in ALL cell lines. EVs that carry this miRNA promote various aspects of leukemic cell behavior, including proliferation, migration, and invasion, while simultaneously inhibiting apoptotic processes. This suggests a pivotal role for miR-181b-5p in enhancing the survival and aggressive characteristics of ALL cells [108]. Similarly, EVs derived from patients with pediatric ALL (pALL) contain miR-181a, which upregulates several proliferation and survival genes, such as proliferating cell nuclear antigen (PCNA), Ki-67, myeloid cell leukemia-1 (MCL-1), and B-cell lymphoma 2 (BCL2). In vitro experiments have demonstrated that specific inhibition of miR-181a can reverse the proliferation induced by EVs, indicating its potential as a valuable therapeutic target in the treatment of ALL [109].

In adult T-cell leukemia/lymphoma (ATL), which is associated with infection by the human T-lymphotropic virus type 1 (HTLV-1), infected lymphocytes release EVs containing viral Tax proteins, pro-inflammatory mediators, and viral mRNA transcripts [110]. These EVs enhance the survival of leukemia cells by inhibiting the expression of Fas, a receptor that mediates apoptosis, through the activation of the AKT signaling pathway. This anti-apoptotic effect is contingent upon the presence of cFLIP (cellular FLICE-like inhibitory protein), a protein that inhibits apoptosis signaling, further underscoring the role of EVs in promoting cell survival in leukemia [111].

Moreover, leukemia-derived EVs exhibiting a stem cell phenotype not only enhance their own proliferation and survival but also bolster the survival and growth of other malignant cells [96].This creates a self-perpetuating signaling loop that contributes to chemoresistance in leukemia. Factors like fibroblast growth factor-2 (FGF2) and HMG-CoA reductase play crucial roles in this process, as increased cholesterol production supports the formation of cell membranes in rapidly dividing cells, facilitating their growth [112].

In addition, the activation of the STAT3 signaling pathway by specific microRNAs, such as miRNA-1246, involves complex cellular and molecular mechanisms that enhance the malignant behavior and aggressiveness of leukemia. MiR-1246 can be packaged into EVs or released directly by leukemia cells, facilitating its uptake into neighboring cells through endocytosis. Once inside the cell, miR-1246 associates with the RNA-induced silencing complex (RISC), where it binds to complementary sequences in the 3′ untranslated region (UTR) of target mRNAs regulating the STAT3 pathway, typically inhibiting negative regulators like SOCS proteins and promoting STAT3 activation [113]. Upon stimulation by cytokines like IL-6, JAKs associated with cytokine receptors phosphorylate STAT3, enabling its dimerization and translocation to the nucleus, where activated STAT3 binds to specific DNA response elements, inducing the transcription of oncogenic genes involved in cell proliferation, survival, and angiogenesis, such as BCL2, MCL-1, and cyclin D1. This process not only reinforces the aggressive characteristics of leukemia cells but also establishes a feedback loop where increased STAT3 activity promotes higher expression of miR-1246, creating a self-sustaining cycle that maintains elevated levels of both miR-1246 and STAT3 activation [114]. Ultimately, this enhances the survival and resistance of leukemia cells to therapeutic interventions, highlighting the potential of targeting this pathway in developing new treatment strategies for leukemia.

Despite the well-documented pro-leukemia effects of EVs, some studies revealed opposing outcomes that highlight the complexity of their role in leukemia [115–117]. For example, EVs derived from mesenchymal stem cells (MSCs) have been shown to reduce the chemosensitivity of ALL blasts by transferring specific miRNAs, such as miR-10a [118]. Conversely, other findings indicate that MSC-derived EVs can induce apoptosis in leukemia cells by altering the expression of pro-apoptotic proteins like BID and BAX while decreasing the expression of anti-apoptotic proteins like BCL2 [119]. This duality in the action of EVs illustrates the intricate communication network they create in leukemia, where the effects can vary significantly depending on the specific cellular context and the components involved. This complexity underscores the potential for developing targeted therapeutic strategies that can exploit the varying influences of EVs in leukemia treatment [120].

### Effects of EVs on leukemia drug resistance

EVs are critical mediators of chemotherapy resistance in leukemia, acting through complex interactions between leukemia cells and the BMM. These vesicles, secreted by both leukemia cells and cells within the BMM, carry a variety of bioactive molecules that significantly influence the behavior of recipient cells. This intercellular communication allows leukemia cells to adapt to and resist chemotherapy, promoting their survival and proliferation. The mechanisms involved in this process are multifaceted, involving the transfer of resistance-conferring molecules, activation of key survival pathways, and modifications to the leukemia-supporting environment [81, 121].

One of the primary mechanisms by which EVs mediate chemotherapy resistance is through the horizontal transfer of drug resistance proteins and genetic material from chemotherapy-resistant leukemia cells to their chemotherapy-sensitive counterparts [122]. Resistant leukemia cells release EVs containing specific proteins, such as ATP-binding cassette (ABC) transporters like P-glycoprotein (MDR1). These proteins function as efflux pumps, actively transporting chemotherapeutic agents out of cells, thus reducing their cytotoxic effects. When sensitive leukemia cells take up these EVs, they begin expressing these resistance proteins, which enables them to pump out drugs like doxorubicin or vincristine, thereby reducing the drug’s intracellular concentration and promoting survival. This transfer of resistance traits leads to a heterogeneous population of leukemia cells, where even those previously vulnerable to chemotherapy become resistant, complicating treatment outcomes [123].

In addition to directly transferring drug-resistant proteins, EVs carry other molecular cargo that activates signaling pathways central to leukemia cell survival. Galectin-3, a lectin protein packaged into EVs by BM MSCs, plays a significant role in activating key signaling cascades that contribute to chemotherapy resistance. Galectin-3 has been shown to engage the Wnt/β-catenin signaling pathway in leukemia cells [124]. The Wnt/β-catenin pathway is crucial for regulating cell proliferation, differentiation, and survival, making it an attractive target for cancer cells. Once activated, β-catenin translocates into the nucleus, where it promotes the transcription of genes that enhance leukemia cell growth and survival, even in the presence of chemotherapy [125]. This pathway is frequently dysregulated in leukemia and its activation through EVs-mediated Galectin-3 delivery further fortifies the leukemia cells’ resistance to treatment [124]. Pro-inflammatory cytokines, which can create a more favorable microenvironment for leukemia cells. In pre-B-ALL, Galectin-3-containing EVs have also been shown to activate the nuclear factor kappa-light-chain-enhancer of activated B cells (NF-κB) signaling pathway [126]. NF-κB is a key transcription factor involved in regulating immune responses, inflammation, and cell survival. In the context of leukemia, NF-κB activation leads to the transcription of anti-apoptotic genes, such as BCL-2 and BCL-XL, which help leukemia cells evade apoptosis, induced by chemotherapeutic agents. The sustained activation of the NF-κB pathway in leukemia cells, triggered by EVs-mediated Galectin-3 transfer, thus plays a crucial role in their resistance to chemotherapy, making it more challenging to induce remission [127].

Another key aspect of Evs-mediated drug resistance in ALL is the transfer of miRNAs, which regulate gene expression in recipient cells. Extracellular vesicle miRNAs (EV-miRNAs) can modulate pathways involved in cell survival, apoptosis, and drug metabolism [128]. For instance, miR-181a, commonly observed in EVs from ALL cells, has been shown to confer resistance to glucocorticoids, a class of drugs widely used in ALL therapy. miR-181a achieves this by downregulating pro-apoptotic proteins, such as BCL-2-interacting mediator of cell death (BIM), thereby preventing the induction of apoptosis in response to glucocorticoid treatment [129]. Similarly, miR-19 can activate the PI3K/AKT signaling pathway, which regulates cell survival, growth, and metabolism. This activation enhances leukemia cell survival and resistance to chemotherapy by phosphorylating downstream targets that inhibit apoptosis and promote cell proliferation, allowing the leukemia cells to thrive despite the presence of cytotoxic drugs [130]. Likewise, exosomes containing miR-21, an oncogenic miRNA, can suppress tumor suppressor genes like PTEN, promoting leukemia cell survival and resistance to chemotherapy [131].

In addition to these local effects within the bone marrow, EVs facilitate long-range communication between leukemia cells and distant components of the immune system, contributing to immune evasion and systemic resistance. For instance, EVs have been shown to modulate the activity of immune cells, including T cells and dendritic cells, by delivering immunosuppressive molecules like TGF-β and PD-L1. This immune modulation suppresses anti-leukemia immune responses, allowing leukemia cells to escape immune surveillance and persist despite chemotherapy. By altering the immune landscape, EVs further ensure the survival of residual leukemia cells, which can lead to relapse after the initial response to treatment [102].

Another important factor is the role of hypoxia in promoting EVs release. In ALL, rapidly proliferating leukemia cells can create a hypoxic environment, particularly within the bone marrow. This hypoxia stimulates the release of EVs, which carry miRNAs and proteins that contribute to drug resistance. These hypoxia-induced EVs further modulate the local environment to support leukemia cell survival and reduce the efficacy of chemotherapeutic agents [132].

Understanding these mechanisms highlight the critical roles that EVs play in leukemia chemotherapy resistance and emphasizes the need for innovative therapeutic strategies. Targeting EVs production, release, or uptake, as well as the key molecules they transport, presents new opportunities to combat chemotherapy resistance and improve treatment outcomes for leukemia patients [121].

### Implications of EVs in leukemia immunotherapy

EVs have a dual role in leukemia immunotherapy, serving as mediators of immune suppression and as therapeutic tools. Leukemia-derived EVs promote immune evasion by delivering immunosuppressive molecules that impair NK cell function and suppress T-cell activity, enabling leukemia cells to survive [96]. Conversely, immune cell-derived EVs can be engineered to enhance anti-tumor responses, acting as cancer vaccines and targeted drug delivery systems. These therapeutic EVs contain bioactive molecules that interact with the immune system, and ongoing research aims to improve their efficacy through genetic modifications and surface engineering. Further, EVs may serve as biomarkers for monitoring treatment efficacy and predicting patient outcomes [120].

#### EVs in cancer vaccines

EVs have gained considerable attention for vaccine development due to their unique ability to transport and deliver bioactive molecules directly to target cells. This natural delivery system enables EVs to play a critical role in modulating immune responses by presenting antigens to immune cells, making them particularly attractive for designing cancer vaccines [120]. Additionally, EVs can be engineered to carry specific tumor antigens, further enhancing their ability to stimulate robust and targeted immune responses [133].

A promising strategy to improve the efficacy of dendritic cell (DC)-based vaccines involves the use of tumor-derived EVs to deliver antigens directly to DCs [134]. For instance, EVs derived from TGF-β1-silenced leukemia cells (LEXTGF-β1si) have demonstrated improved effectiveness in DC-based leukemia vaccines. In this approach, a lentiviral shRNA vector was employed to silence TGF-β1 expression in leukemia cells, and EVs were subsequently isolated from these modified cells. These EVs were then used to pulse dendritic cells (DCLEX-TGF-β1si), resulting in enhanced DC maturation, immune function, and anti-tumor response. Findings revealed that DCLEX-TGF-β1si significantly boosted CD4+ T-cell proliferation, promoted Th1 cytokine secretion, and induced a robust tumor-specific cytotoxic T lymphocyte (CTL) response in vitro. Additionally, in animal models, the modified DC vaccine (DCLEX-TGF-β1si) effectively inhibited tumor growth compared to control formulations, demonstrating efficacy as both a preventive and therapeutic vaccine [135]. Similarly, another study aimed to improve anti-leukemia immune responses using leukemia cell-derived EVs loaded onto DCs modified to express co-stimulatory molecules CD80 and CD86 (LEX-8086). These molecules are essential for enhancing DC function and boosting immune efficacy. The modified DCs (DCsLEX-8086) were found to significantly enhance DC maturation, improve antigen presentation, and activate CD8+T cells, leading to stronger CTL responses. In animal models, DCsLEX-8086 not only inhibited tumor growth but also increased levels of CD4+ and CD8+T cells, along with M1 macrophages in the tumor microenvironment, while reducing Tregs [136]. Moreover, modifications to EVs derived from leukemia cells, such as downregulating PD-L1 expression using short hairpin RNA, have shown additional promise in leukemia immunotherapy. EVs from PD-L1-silenced leukemia cells (LEXPD-L1si) enhanced DC maturation, stimulated T helper cell proliferation, increased cytokine release, and boosted CTL activity. These results were further validated in vivo, where vaccination with LEXPD-L1si significantly inhibited tumor growth and extended the survival of immunized mice [117]. These studies collectively highlight the essential role of EVs in enhancing dendritic cell-mediated immune responses, while also shaping the broader immune response against tumor cells.

In parallel, EVs derived from immune cells, particularly dendritic cell-derived EVs (Dex), have also shown great potential in leukemia immunotherapy. Dex can be engineered to carry leukemia-specific antigens, proteins, or peptides derived from leukemia cells, making them ideal vehicles for cancer vaccines [137]. When dendritic cells are loaded with these antigens, they generate EVs containing MHC class I and II molecules, co-stimulatory molecules CD80 and CD86, as well as the antigen itself. Once administered to patients, these Dex activate both CD8+ cytotoxic T cells and CD4+ helper T cells, triggering a robust immune response against leukemia cells. As Dex circulate through the body, they interact with T cells, mimicking the antigen-presenting function of their parent DCs and training the immune system to recognize and target leukemia cells. This process holds great potential for improving patient outcomes by inducing a targeted immune response [138]. Unlike tumor-derived EVs, Dex are less likely to carry immunosuppressive molecules like PD-L1 and TGF-β, making them more efficient at inducing an anti-leukemia response. Their natural ability to stimulate the immune system also makes them particularly effective in overcoming the immunosuppressive tumor microenvironment commonly found in leukemia [137].

#### EVs in drug delivery

EVs are emerging as highly promising nanocarriers for drug delivery in cancer therapy, including the treatment of ALL. Their unique properties, such as high biocompatibility, low immunogenicity, and the ability to transport therapeutic molecules directly to target cells, make them ideal for enhancing drug efficacy while minimizing side effects [139]. A major challenge in treating acute leukemia is drug resistance, which often leads to treatment failure or relapse. Traditional chemotherapy does not always reach leukemic cells effectively, and cancer cells can develop mechanisms to resist these treatments [140]. EVs offer a solution by delivering therapeutic agents directly to leukemic cells, enhancing drug effectiveness and minimizing systemic toxicity. Their natural ability to target specific tissues reduces off-target effects and limits damage to healthy cells, which is critical for overcoming drug resistance in ALL [141].

EVs have a high capacity for cellular uptake, allowing them to be easily absorbed by recipient cells. Their membrane-like structure facilitates seamless fusion with target cells, ensuring the efficient release of their therapeutic cargo. This ability to bypass cancer resistance mechanisms further enhances their effectiveness in ALL treatment. Moreover, EVs can carry both hydrophilic and hydrophobic drugs, improving the solubility and stability of cancer therapies that may be less effective when delivered through conventional methods [142].

Another key advantage is their biologic origin, which gives EVs high biocompatibility. Unlike synthetic drug carriers, EVs are less likely to provoke immune responses, enabling longer circulation in the bloodstream. This feature is particularly beneficial for treatments requiring repeated or prolonged administration, as it allows for a controlled and sustained release of therapeutic agents [120].

In combination therapies, EVs demonstrate significant potential by delivering chemotherapy drugs alongside gene-editing tools, such as small interfering RNA (siRNA), to silence drug-resistant genes in cancer cells. This dual capability strengthens their role in personalized medicine, enabling treatments to be tailored to the genetic and molecular characteristics of a patient’s cancer, thereby improving therapeutic outcomes [143].

To further optimize drug delivery, EVs can be engineered to enhance their targeting ability. One approach involves the incorporation of aptamers, short, single-stranded oligonucleotides that bind to specific proteins on cancer cells [144]. For example, AS1411, an aptamer targeting nucleolin (overexpressed in ALL cells), can guide EVs loaded with chemotherapeutic drugs or RNA-based therapies like siRNA and miRNA directly to leukemic cells, potentially overcoming drug resistance [145]. Another strategy is the use of tumor-homing peptides, such as RGD, which have a high affinity for integrins, proteins often overexpressed in tumor cells. RGD-modified EVs have demonstrated increased cytotoxic effects of treatments like doxorubicin by binding to integrins on leukemic cells, enhancing drug delivery and improving treatment outcomes while reducing systemic side effects [141].

EVs can also be modified with antibodies, such as anti-CD19 or anti-CD22, to target B-cell markers overexpressed in B-cell ALL, improving the specificity and efficiency of drug delivery. Other targeting strategies include using folate ligands to bind leukemia cells with overexpressed folate receptors or transferrin to target rapidly dividing cells, enhancing the uptake of EVs by leukemic cells [146].

Beyond targeting cancer cells, EVs can be engineered to engage the immune system. For instance, EVs modified with CD40 ligands can activate dendritic cells, leading to improved T-cell responses and reduced tumor growth in ALL [147]. Similarly, EVs engineered with interleukin-12 (IL-12) can stimulate T cells and NK cells, boosting the immune system’s ability to combat leukemia within the tumor microenvironment [148].

While EVs offer great potential in improving ALL treatment by overcoming drug resistance and reducing systemic side effects, several challenges remain in translating these therapies into clinical practice [149]. Future advancements in EVs engineering and the development of hybrid systems that combine EVs with synthetic materials may help address these challenges, unlocking the full therapeutic potential of EVs as a versatile drug delivery platform [142].

#### Role of EVs as biomarkers

EVs hold significant promise as vital biomarkers in the management of ALL, providing valuable diagnostic, prognostic, and therapeutic insights. Released by leukemic cells and their surrounding tumor microenvironment, these small vesicles carry biomolecules that reflect the molecular characteristics of leukemia. This unique property makes exosomes powerful tools for diagnosing the disease, monitoring its progression, and assessing treatment responses [150, 151].

In the context of ALL, EVs contain leukemia-specific proteins, such as CD19 and CD22, commonly associated with B-cell precursor ALL (BCP-ALL). Detecting these proteins in blood or other body fluids offers a non-invasive method for early leukemia detection, reducing the need for invasive procedures like bone marrow biopsies. This approach facilitates timely interventions, ultimately improving patient management and outcomes [152].

Early detection is crucial for determining appropriate treatment strategies [153]. EVs can identify leukemia before clinical symptoms manifest or the disease progresses. Monitoring EVs levels and their molecular composition can reveal leukemic changes at their earliest stages. For instance, circulating EV-miRNAs, such as miR-128, miR-223, and let-7b, can differentiate ALL from other leukemia forms and distinguish between subtypes like B-ALL and T-ALL [154].

Beyond their diagnostic potential, EVs provide important prognostic information. Specific EV-miRNAs, including miR-155, miR-150, and miR-1246, have been identified as key biomarkers for disease progression and treatment outcomes in acute leukemia [155]. Elevated levels of miR-155 are associated with more aggressive forms of leukemia and poorer prognosis, as it promotes cell proliferation by targeting the SHIP1 tumor suppressor gene, which regulates a crucial signaling pathway for cell growth [156]. Conversely, the downregulation of miR-150 disrupts normal regulatory mechanisms, contributing to tumor progression by allowing the overexpression of c-Myb, a transcription factor critical for hematopoiesis [157].

Additionally, EVs from patients with ALL contain immunosuppressive molecules that influence immune T-cell functions, indicating their potential as biomarkers for assessing cancer stage. These Evs play a role in modulating the bone marrow microenvironment, tumor cell apoptosis, angiogenesis, and immune evasion mechanisms, significantly impacting leukemia onset and progression [102].

Despite the potential of Evs as biomarkers, challenges remain in standardizing their isolation and analysis for clinical practice. Addressing these issues is crucial for fully integrating EVs into routine diagnostics and patient care, ensuring their full potential is realized in managing ALL [158].

### Limitations of EVs clinical applications

While EVs hold significant promise for the diagnosis and treatment of leukemia, several limitations need to be addressed to fully harness their clinical potential. One of the main challenges is the isolation and purification of EVs. Despite being obtainable from various biologic fluids like blood, urine, and saliva, extracting EVs in sufficient quantities while maintaining their integrity is difficult [159]. Current isolation techniques, including ultracentrifugation and size-exclusion chromatography, are often time-consuming and may yield impure or heterogeneous populations. Contaminants such as proteins and lipids can compromise the quality and therapeutic efficacy of the EVs, necessitating standardized protocols for their isolation and ensuring consistent, reproducible results in clinical applications [160].

Another major limitation is the heterogeneity of EVs. These EVs vary in size, content, and surface markers depending on their cellular origin and the state of the cells from which they are derived. This variability makes it challenging to predict their behavior and therapeutic outcomes in clinical settings. Universal criteria for characterizing exosomes, including their size, molecular content, and functional properties, are still lacking and are critical for advancing their therapeutic use [161].

Immunogenicity is also a key concern in exosomal applications. While autologous EVs derived from the patient’s own cells generally have low immunogenicity, donor-derived or engineered EVs may trigger undesirable immune responses. Variations in immunogenicity between different patient populations and EVs batches can lead to inconsistent therapeutic outcomes, complicating the treatment process [162]. Ensuring EVs can effectively target specific cells, such as leukemic or immune cells, while maintaining their stability and functionality is essential for their successful clinical use [163].

Additionally, the tumor microenvironment poses significant obstacles to the delivery and efficacy of EV-based therapies. In leukemia, the microenvironment is often highly immunosuppressive, limiting the effectiveness of EV-based immunotherapies. Tumor-derived EVs can carry immunosuppressive molecules that counteract therapeutic efforts, further complicating their use. A deeper understanding of how EVs interact with the tumor microenvironment is necessary to enhance their therapeutic impact [164].

Finally, regulatory and safety concerns present challenges in the clinical application of EVs. As biologic therapies, EV-based treatments are subject to strict regulatory requirements, and comprehensive studies are needed to evaluate their safety and efficacy. This regulatory scrutiny can slow down development and increase costs, making it more difficult to bring these therapies to market. Furthermore, the production, storage, and logistical requirements of EVs, particularly maintaining their stability, can also lead to higher costs, making it challenging to scale and implement these treatments in clinical practice, especially in resource-limited settings [165]. Overcoming these limitations is critical for realizing the full potential of EV-based therapies in treating leukemia and other diseases [166].

## Conclusion

EVs play critical roles in the pathogenesis of ALL by promoting tumor growth, drug resistance, and immune evasion. They contribute to disease progression by facilitating angiogenesis, remodeling the bone marrow microenvironment, and transferring oncogenic molecules like miRNAs. Notably, EVs also offer promise as diagnostic and prognostic biomarkers due to their ability to carry disease-specific molecular signatures, potentially enabling less invasive monitoring and personalized treatment approaches. However, challenges such as EV heterogeneity and technical limitations in isolation must be addressed before they can be fully utilized in clinical settings. Their dual role in leukemia pathogenesis and as potential biomarkers makes EVs a promising target for future research and therapeutic interventions.

## Data Availability

The datasets used and/or analyzed during the current study are available from the corresponding author on reasonable request.
